# App-based self-management program for people dealing with fatigue and trouble concentrating (DiEgO): protocol for participatory development, pilot testing, and qualitative process evaluation

**DOI:** 10.1186/s40814-026-01821-x

**Published:** 2026-04-22

**Authors:** Tim Schmachtenberg, Nadja Wegner, Imke Schwalm, Karla Clasen, Katharina Vieth, Tim Riester, Nicole Schmidt, Viktoria Lampe, Zehra Bilgen, Aisha Cook, Frank Müller, Eva Maria Noack, Alexandra Dopfer-Jablonka, Christine Happle, Sandra Steffens, Georg Behrens, Eva Hummers, Andrea Stölting, Torge-Christian Wittke

**Affiliations:** 1 Department of General Practice, University Medical Center Göttingen, Humboldtallee 38, Göttingen, 37073 Germany; 2 Department of Rheumatology and Immunology, Hannover Medical School, Carl-Neuberg-Str. 1, Hannover, 30625 Germany; 3 Regional Cooperative Rheumatism Centre Lower Saxony e.V., Karl-Wiechert-Allee 3, Hannover, 30625 Germany; 4 TIMMCOOK Occupational Therapy GmbH, Berenbosteler Straße 76B, Garbsen, 30823 Germany; 5 Department of Medical Education, Hannover Medical School, Carl-Neuberg-Str. 1, Hannover, 30625 Germany

**Keywords:** Intervention development, mHealth, Occupational therapy, Exercise therapy, Fatigue, Trouble concentrating, Post-COVID syndrome, Participation, Qualitative study

## Abstract

**Background:**

Fatigue is a common symptom in both post-COVID syndrome (PCS) and chronic inflammatory rheumatic conditions, associated with physical, cognitive, and mental impairments that reduce quality of life. Evidence-based management approaches remain limited. The DiEgO study addresses this gap by developing an interactive self-management and support application for people with fatigue and trouble concentrating.

**Methods/design:**

This study combines a participatory research approach with qualitative methods (content analyses, grounded theory). A development team comprising PCS individuals, occupational therapists, and academic researchers will collaboratively develop the intervention through three iterative cycles. The developed app will then be tested by 20–30 people with rheumatic-disease-related fatigue, assigned to either supported use (with occupational therapy) or independent use. Evaluation includes qualitative interviews with pilot participants to assess usability, feasibility, and acceptability. In addition, the participatory development process will be reflexively evaluated through participant observation and interviews with development team members.

**Discussion:**

The DiEgO study will develop an mHealth self-management application for people with fatigue and trouble concentrating. The participatory design involving affected individuals and occupational therapists throughout the development process enhances user-centeredness and real-world applicability. By providing flexible, low-threshold access through an open-source platform, this study may expand accessible self-management support for individuals with post-COVID syndrome and rheumatic conditions.

## Background

Fatigue (FTG) is a common symptom of chronic conditions, occurring in about 40–90% of all cancer patients, 60% of people with rheumatic conditions or multiple sclerosis (MS) [1], and 45% of people with post-COVID syndrome (PCS) [2]. FTG is characterized by persistent, excessive tiredness and exhaustion that occurs independently of prior exertion and cannot be alleviated by rest periods [1]. People with FTG are often affected by physical symptoms such as a lack of strength and energy, cognitive complaints such as trouble concentrating (TC), and mental problems such as listlessness [1], negatively affecting their quality of life, daily coping, and work ability [3].

Currently, there are no treatment options available that target the underlying causes of FTG; consequently, care focuses primarily on symptom management [4]. However, the range of evidence-based and specialized symptom-oriented approaches remains inadequate given the conditions’ complexity [5, 6], creating challenges for both affected individuals and their healthcare providers. Studies suggest that occupational therapy can be a suitable approach to improve symptom management for people with FTG and TC [7–9]. Accordingly, some PCS care guidelines recommend occupational therapy as part of a multimodal treatment approach [10, 11].

The COVID-19 pandemic accelerated demand for remote healthcare delivery, including increased adaption of video consultations [12], which German healthcare providers have been able to use flexibly since 2022 [13]. Furthermore, to foster remote care, various mHealth applications have been introduced and are covered through the statutory health insurance system. However, an online search on available products revealed a lack of scientifically evaluated, non-commercial digital approaches for occupational therapy treatment of people with FTG and TC. This gap is particularly relevant given mobility limitations and ongoing access barriers faced by individuals with chronic fatigue conditions [14].

The DiEgO (Digital Occupational Therapy for FTG and TC) study aims to address this gap through the participatory development of an mHealth application. Researchers and occupational therapists collaborate with individuals experiencing FTG and/or TC to develop evidence-based content for self-management support, which is then piloted among patients with FTG and/or TC from PCS and inflammatory rheumatic diseases (IRD). Building on the predecessor project ErgoLoCo (Occupational Therapy for Long COVID) [15–17], which demonstrated promising results in a first unpowered pilot randomized controlled trial [16], but also revealed a need for more interactive rather than a fixed video-only format [18], DiEgO is conceptualized as a self-paced, interactive intervention enabling users to learn and implement occupational and exercise self-management strategies, with flexible options for independent use or professional support and informed by people experiencing FTG and TC first-hand.

Participatory approaches can yield intervention that better align with users’ needs and expectations; it entails several challenges. People with persistent fatigue experience fluctuating capacity to engage may require flexible forms of participation that accommodate symptom variability. Healthcare hierarchies between patients and professionals can constrain collaborative decision-making if unaddressed [19]. The intangible nature of software adds further complexity to participatory development approaches as they may remain abstract until implemented, making it difficult to envision functionality or assess usability without prototypes and iterative testing [20].

Given these complexities, this project aim to (a) collaboratively develop an interactive, self-paced mHealth application that enables people experiencing FTG and trouble concentrating, (b) conduct a pilot study and assess usability, feasibility, and acceptability, and (c) examine the participatory development process itself, evaluating whether and how meaningful co-researcher involvement can be achieved when developing digital health technologies with individuals who have chronic conditions.

## Methods/design

### Study design

This 18-month project uses a participatory research approach for the intervention development [21, 22] followed by pilot test and process evaluation using qualitative research methods. Data are collected through participant observation during workshops, semi-structured interviews, and analysis of written feedback. Qualitative content analysis will be used to evaluate usability, feasibility, and acceptability, while grounded theory is applied to analyze and reflect the participatory development process [23, 24]. The application is developed using the eHealth platform CIAS-EU (Computerized Intervention Authoring System) [25], an open-source eHealth development platform that enables collaborative creation of interactive digital interventions without requiring programming skills.

### Study setting

DiEgO is conducted in the German federal state of Lower Saxony, involving academic and community-based healthcare settings. Study centers include the Department of General Practice at University Medical Center Göttingen (UMG) and the Regional Cooperative Rheumatism Centre of Lower Saxony, with additional involvement from researchers at the Department of Rheumatology and Immunology at Hannover Medical School (MHH).

### Participatory development

#### Conceptual framework

The intervention follows a participatory research approach, positioning PCS individuals as experiential co-researchers and occupational therapists as practice co-researchers rather than study subjects [26]. Participatory research centers on understanding lived experiences and translating them into actionable solutions [26]. Achieving this requires co-researcher involvement in nearly the entire research and decision-making process, from formulating research questions and defining goals, selecting methods, conducting research, and publishing results—rather than providing input when asked [27, 28].

DiEgO uses a participatory approach because it grounds development in lived experience [27]. This is particularly important when working with chronically ill populations creating intangible digital products, where theoretical assumptions may diverge substantially from actual needs and usage patterns and may undermine acceptability, suitability, and feasibility [20, 29].

In recent years, several models have been proposed to assess level of participation in research [22, 26, 27]. This study uses Cornwall’s level of “co-operation” [22], where co-researchers help determine research topic and direction. However, overall responsibility for the research process remains with the academic researchers [27]. This mid-level model balances meaningful involvement with pragmatic constraints, by including perspectives, experiences, and needs of individuals from DiEgO’s two target groups while protecting people with chronic health complaints from overburdening and ensuring timely development within temporal and personnel framework of the project.

**Fig. 1:**
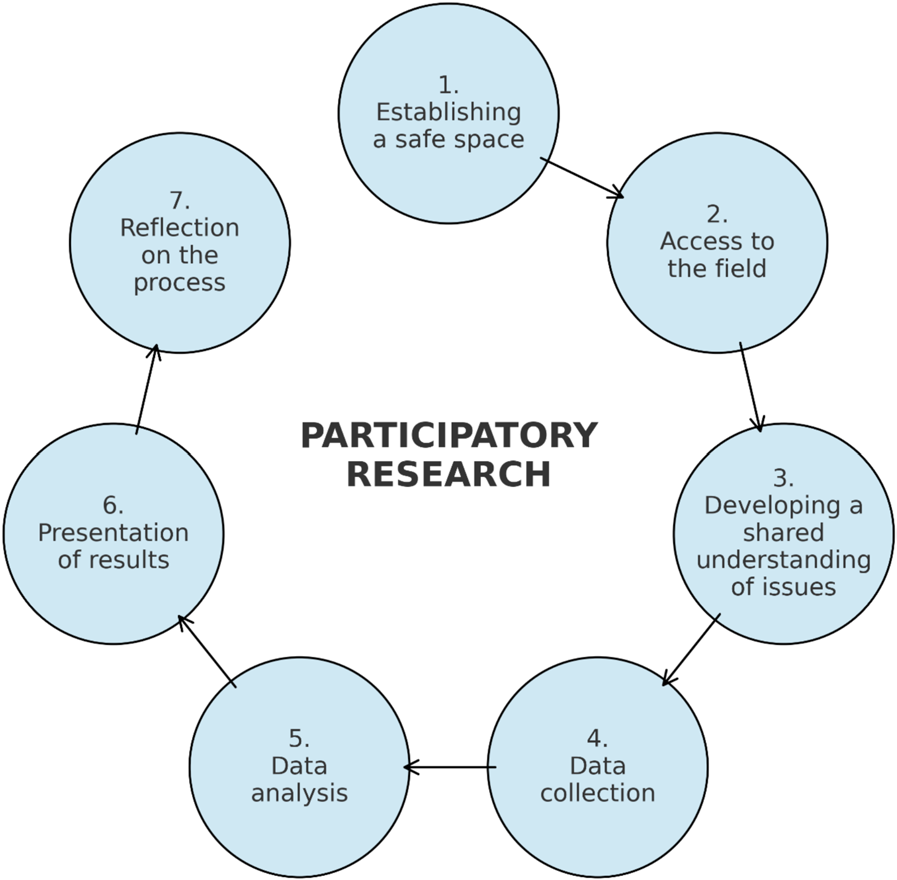
Seven components of the participatory research process model by Hartung et al. [21]

The research process of this study is based on the participatory research process model by Hartung et al. [21], whose components are presented in Fig. 1.

#### eHealth platform CIAS-EU

**Fig. 2:**
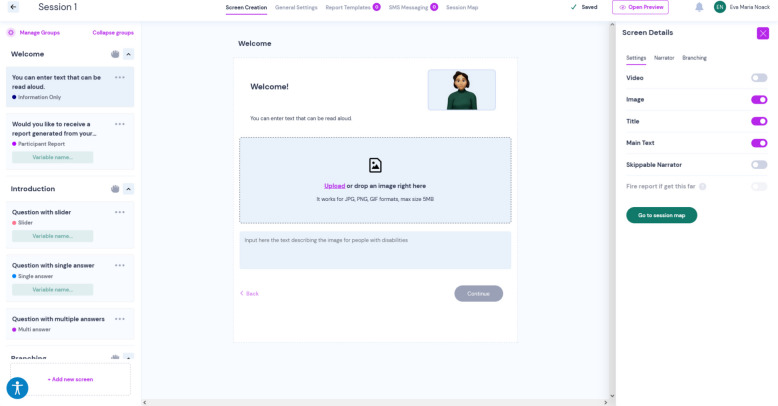
Graphical user interface of the authoring environment for the design of the app

**Fig. 3:**
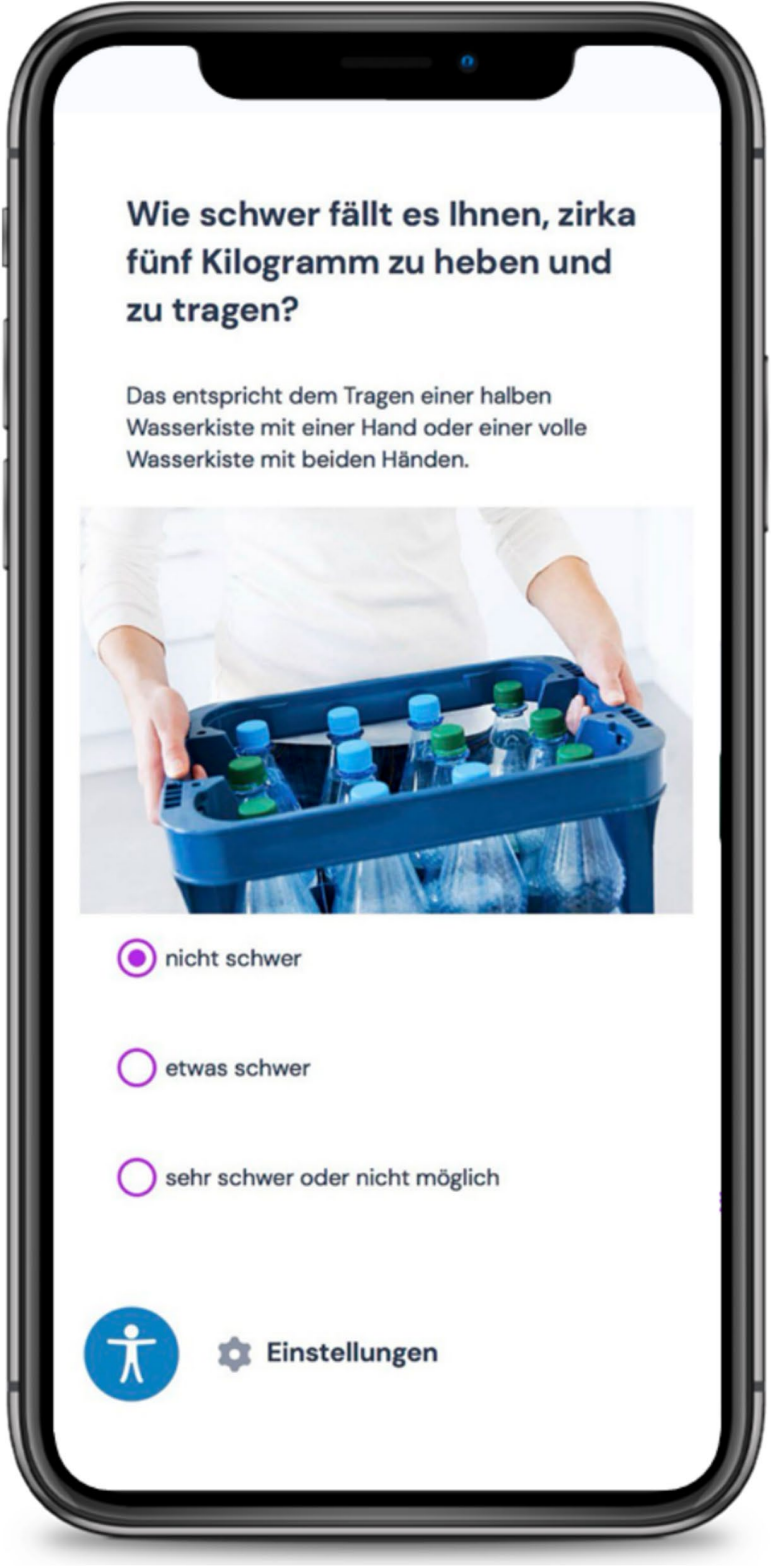
Screen of an intervention as seen by participants using their smartphones

CIAS is a non-commercial, open-source platform for creating digital health interventions. CIAS provides a graphical user interface for designing digital interventions (see Fig. 2), allowing researchers to draft, edit, share, and utilize interactive and multimedia interventions for research projects without programming knowledge. The interventions can be organized into session-based content tailored to participants. Images and videos can be embedded, and content can be narrated by a virtual character. As no technical expertise is required, interventions can be co-creatively developed with user of various backgrounds and experience levels. Interventions are delivered to participants as cross-platform web apps (Fig. 3).

Originally developed at Michigan State University (MSU), CIAS-EU (www.cias-app.eu) was adapted for European data protection standards and is hosted on GDPR-compliant infrastructure at Society for Scientific Data Processing mbH Göttingen (GWDG) (Noack EM, Ondersma SJ, Müller F: Overcoming barriers in digital health intervention development: introducing the no-code platform CIAS-EU, in preparation).

### Project timeline

**Fig. 4:**
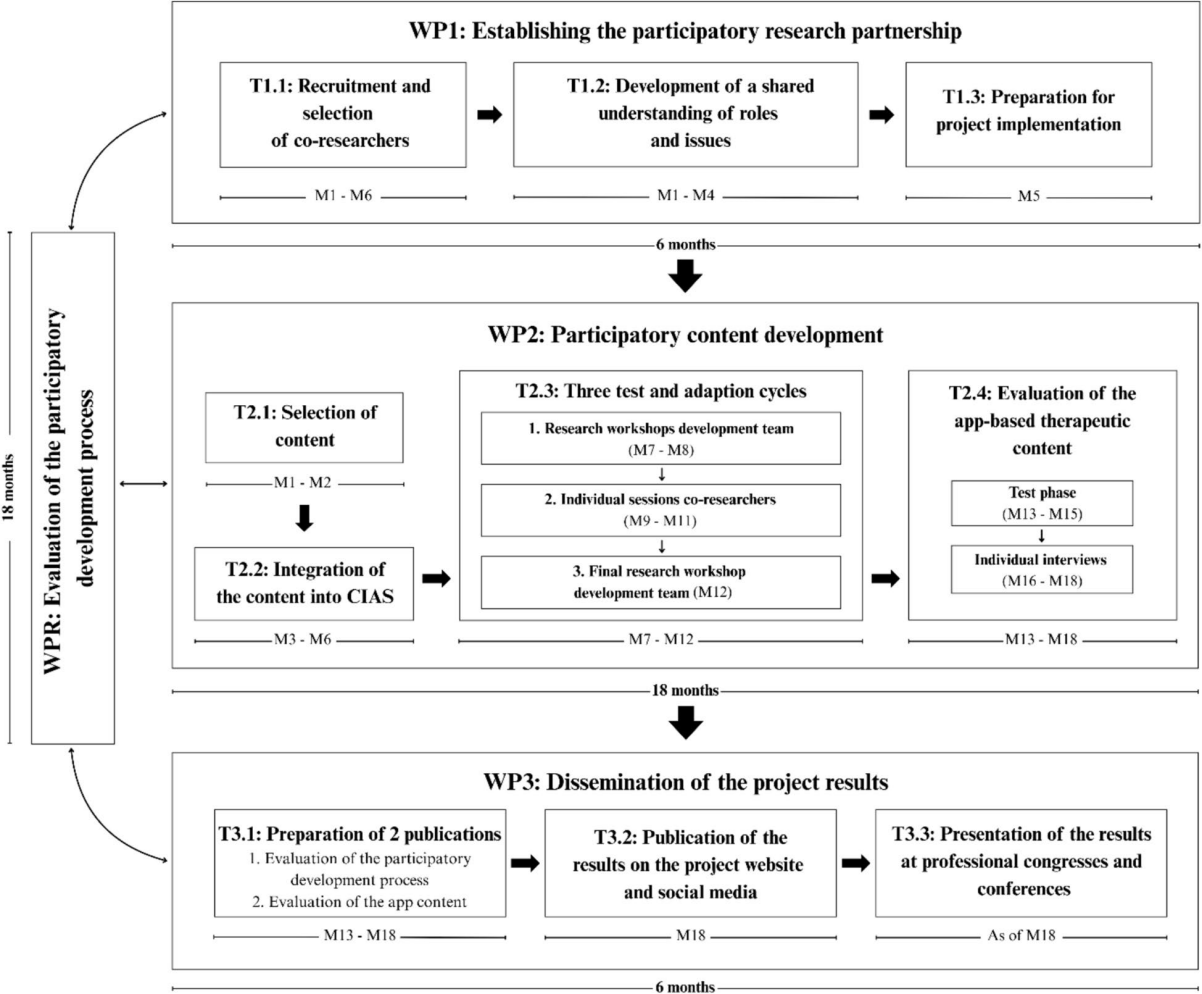
Flowchart of the research process of DiEgO

DiEgO involves three main work packages: establishing the participatory research partnership, participatory content development, and dissemination of the project results. Figure 4 provides a detailed timeline and an overview of all main and sub-work packages of this project.

#### WP1: establishing the participatory research partnership

The first 6 months focus on building a collaborative research partnership. First, individuals with FTG and/or TC and occupational therapists with experience in digitally supported occupational therapy are recruited and integrated into DiEgO’s development team (T1.1, M1–M6). Introductory sessions during this phase build trust, clarify expectations regarding participation, and identify co-researchers’ concerns and needs. Building on these initial sessions, the team conducts a workshop to establish shared role definitions and develop mutual problem understanding. This workshop identifies topics and questions relevant to co-researchers’ daily lives and occupational therapy practice, ensuring that subsequent development addresses real-world needs (T1.2, M1–M4).

The goal of the third task (T1.3) is to resolve key organizational, methodological, and technical questions, enabling the start of intervention development. During a research workshop, the content and timeline of further work packages and the methodological approach and task distribution for each development and analysis step will be jointly determined. Additionally, the co-researchers will be introduced to the eHealth platform CIAS-EU.

#### WP2: participatory content development

Application development follows an iterative approach with multiple cycles of creation, testing, and refinement [21]. The process begins with academic researchers compiling elements perceived as feasible and helpful by participants from the predecessor ErgoLoCo project [18]. This involves analyzing qualitative interviews and survey results of ErgoLoCo participants regarding feasibility and usefulness of specific content elements for people with FTG and/or TC, as well as conducting targeted literature searches on other occupational therapy approaches in FTG management. These elements inform the further participatory development phases.

Following, selected elements are transferred to the CIAS-EU platform by academic researchers. An initial application structure is developed during an interdisciplinary workshop involving academic researchers from different professional backgrounds. Once the foundational structure is established in CIAS-EU, the collaborative refinement process begins through three iterative test-and-adaption cycles, where people with FTG and/or TC, occupational therapists, and academic researchers cooperatively refine the content to enhance feasibility and alignment with the lived experiences. This process will consist of 4–5 research workshops and two test sessions for each co-researcher with FTG and/or TC. The research workshops are scheduled for approximately 60 to a maximum of 90 min, and the test sessions for 45 to 60 min. To accommodate participants’ fatigue, breaks will be offered during all sessions; 1–2 scheduled breaks are planned for the research workshops. In addition, co-researchers will have the opportunity to turn off their cameras at any time and to leave the meeting temporarily or permanently. All research workshops and test sessions will be conducted using the teleconference software Zoom (Zoom Communications Inc., San José, CA) and will be recorded, transcribed, and analyzed by academic researchers.

##### Three test and adaptation cycles for the participatory development of the DiEgO app

In the first cycle, three research workshops will be conducted where academic researchers will present the app structure and the intervention elements in CIAS-EU, gather feedback from the other team members, and collect suggestions for improvements. To prevent the initial app structure drafted by the academic researchers from unduly constraining the influence of the co-researchers, the proposed app structure will be openly discussed with the co-researchers during the first research workshop, giving them the opportunity to make fundamental adjustments. Such fundamental adjustments are possible with manageable effort by the researchers due to the flexible structure of the intervention drafts on the CIAS-EU platform, which can be adapted using simple inputs and formulas. Based on the co-researchers’ suggestions, the academic researchers will then make adjustments to the app structure and content. Co-researchers validate the revised version before proceeding to the next cycle. For this purpose, the changes made will be presented to the co-researchers in the following research workshop and then discussed. During these discussions, an attempt will be made to reach a consensus by working in real-time on a shared screen on the contents of DiEgO in CIAS-EU until all participants agree on the solution [23]. If the co-researchers’ expectations cannot be met within the project’s constraints, we will first attempt to adapt the framework of this project to the co-researchers’ needs. For this purpose, we have additional personnel and technical resources available, and we can use alternative participatory and qualitative methods. If the expectations still cannot be met, we will communicate this transparently to the co-researchers and point out the project’s constraints. We will document any requests that go beyond these constraints and take them into account when planning and applying for future participatory research projects.

The second cycle moves to contextualized testing of preliminary app versions: app elements are assigned to occupational therapist/PCS participant pairs who conduct two in-depth test sessions each, ensuring all elements receive evaluation. To maintain overview of the complete application, individuals with PCS subsequently explore remaining content independently at their own pace, providing written feedback. Co-researcher, unable to participate in group and individual sessions due to the severity of PCS, will test selected intervention parts independently and document to what extent the contents are feasible and helpful. To support the co-researchers in this process, they will receive a template with several open-ended questions to guide them in providing their feedback. If they have difficulties with the independent testing or providing written feedback, they can contact the academic researchers by phone, email, or during a Zoom meeting. Participants with severe PCS symptoms will also be recommended to seek support from their caregiver partners (when applicable) if they are unable to test the app’s content or provide written feedback on their own. Additionally, they will be offered a telephone interview with an academic researcher as an alternative if providing written feedback is difficult due to fatigue and/or trouble concentrating. Academic researchers will evaluate all recorded sessions and written feedback and make app refinements. If the individuals with PCS have conflicting requests, a discussion will be held first among the academic researchers and then with the co-researchers. The solution proposed by the academic researchers will be discussed and a consensus decision will be sought [23]. If this is not possible, a vote will be taken according to the majority principle.

The third cycle concludes with a workshop where co-researchers review and provide feedback on the adjusted application. Academic researchers incorporate this input to finalize the pilot version, which all development team members validate before testing begins with individuals from rheumatology practices.

##### Evaluation of the app-based therapeutic content

Following collaborative development, the DiEgO app undergoes pilot testing with 20–30 individuals experiencing FTG due to IRD who were not involved in the previous development process. The aim is to investigate whether the app content could also be helpful for people with FTG and/or TC and other underlying conditions besides PCS. After personal briefing and obtaining written consent, participants will be assigned to two test groups. Both groups will test the same app components to gather comparable qualitative feedback from two likely real-world use scenarios: one group will test the DiEgO content with an occupational therapist, while the other group will test the content without an occupational therapist. This approach is intended to provide initial insights into the extent to which the app can be used without occupational therapy support and where support from an occupational therapist is beneficial. These findings will serve as the basis for designing a follow-up study in which the efficacy of the app’s content will be systematically assessed. No outcome measures will be evaluated as part of this evaluation; it is merely a qualitative evaluation of the app’s experience from the perspective of people with FTG and/or TC.

Group 1 will test the DiEgO app with occupational therapist support through two digital individual sessions (via Zoom). In these two sessions, the therapists will give the patients a brief introduction to the program and the content of the individual intervention elements and will conduct the exercises and a reflection on the session together with them. During these sessions, patients and therapists will document their thoughts on the intervention contents. Subsequently, patients will independently test further intervention elements and provide written feedback. Test group 2 will test the individual intervention elements without therapeutic support. To facilitate this, patients will receive temporary access to the DiEgO app. They will be asked to document their thoughts on the app and tested content and send their feedback to the academic researchers. At the end of the 3-month testing phase (M13–M15), feedback from both groups will be evaluated by academic researchers and prepared for CIAS-EU implementation. The division of the evaluation cohort into two groups allows an exploratory comparison between solo users and patients who receive occupational therapy support when working with the app’s content. This approach enables conclusions regarding the extent to which the DiEgO app and its therapeutic content meet the needs of the two patient groups and what additional adjustments are necessary for maximizing benefits for both groups.

To evaluate the content of the DiEgO app, academic researchers will conduct individual interviews with 12 to 15 patients (from both test groups) following the tests. These interviews will focus on acceptability, feasibility, suitability, integration into daily life, user-friendliness, and usefulness. Recorded interviews will be transcribed and analyzed by the academic researchers. Interview participants will then receive a document with processed results for validation. Based on change requests expressed by patients and therapists during testing and evaluation of intervention elements, the contents of the DiEgO app will be revised one last time by academic researchers. Afterwards, the final version of the app will be presented to the development team and released following verbal and written feedback from the co-researchers.

#### WP3: dissemination of the project results

Project results are disseminated to multiple audiences, including individuals affected by FTG and/or TC, relatives, healthcare providers, and researchers through various channels. Beyond this study protocol, two peer-reviewed manuscripts will be prepared: one examining and reflecting the participatory development process, another focusing on the evaluation of the usability and acceptability of the app. Both manuscripts are primarily authored by academic researchers, validated by co-researchers and pilot participants before submission with co-researchers invited to contribute to results and manuscript preparation. Following project completion, results and application information are published on a dedicated website and shared via social media channels. Academic researchers present findings at professional conferences, ensuring both scientific and public dissemination of this participatory research approach and its outcomes.

## WPR: evaluation of the participatory development process

To critically examine the participatory approach itself, an independent researcher (ISc) conducts a reflexive evaluation throughout the project, assessing how expectations regarding roles, involvement, and collaborative decision-making evolve across the research team. The results of this evaluation are expected to provide insights for future participatory studies focused on developing digital interventions and how co-researchers, particularly individuals with chronic conditions, can be better included in these research projects.

Data collection begins during introductory sessions, where development team members are asked, using a guideline with open-ended questions, about their motivations for participation, their hopes and goals for the project, and their expectations regarding collaboration and application development. Throughout the development workshop, the independent researcher conducts participant observation documenting team interactions, role negotiations, group dynamics, and how co-researchers engage with technical (non-human) actors such as Zoom or CIAS-EU. The observing person will introduce themselves to the development team and disclose their role, but will not actively participate in discussions (passive observation) [30]. Structured observation protocols are developed with methodological experts at the Department of General Practice who are not involved in the DiEgO research project.

Following the development phase, individual interviews will be conducted with academic, experiential, and practice researchers from the development team. In these interviews, participants are prompted to reflect on how their expectations regarding app development and involvement were met or changed during the research process.

### Participants

#### Development team

**Fig. 5:**
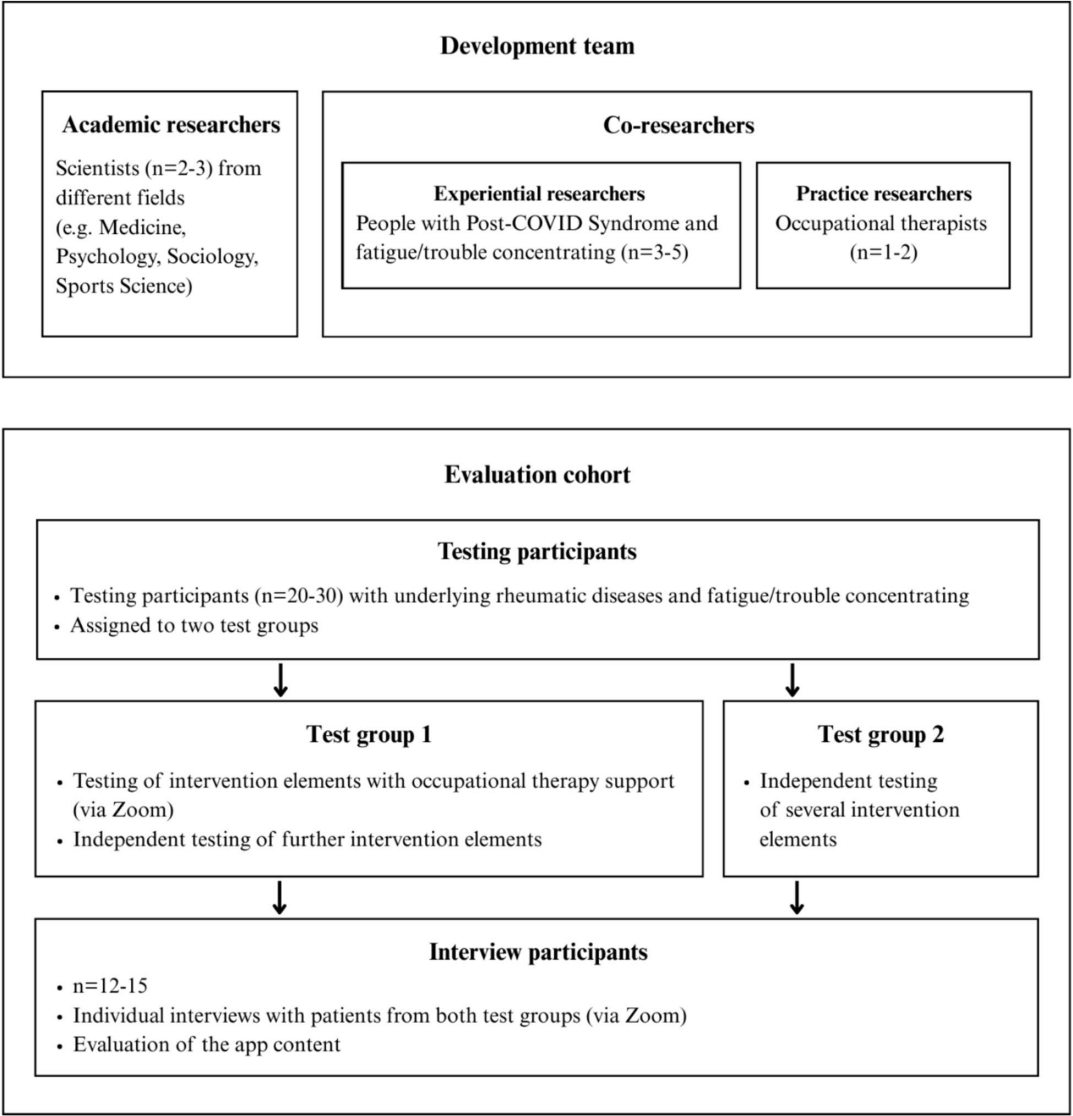
Flowchart of the involved groups

The development team will consist of 3–5 individuals with PCS with interest in the joint development of a self-management app, 1–2 occupational therapists, and 2–3 academic researchers (Fig. 5). Participants with severe symptoms unable to participate in workshops are invited to provide written feedback on preliminary app versions during the development process.

##### Eligibility criteria

The following criteria constitute the prerequisites for including individuals with PCS in the development team: (1) minimum age of 18 years; (2) self-reported PCS condition, defined as “health complaints persisting or newly occurring beyond the acute phase of a SARS-CoV-2 infection, lasting four weeks” [31]; (3) subjectively experienced symptoms of FTG and/or TC; (4) internet access; (5) an internet-enabled device; and (6) written consent to participate in the study. Individuals unable to participate in video conferences or provide written feedback due to the severity of their symptoms or actuation problems will not be selected for the development team for practical and ethical reasons.

Eligibility criteria for occupational therapists are (1) self-reported practical experience in treating patients with PCS and FTG and/or TC; (2) internet access; (3) an internet-enabled device; and (4) written consent to participate in the study.

##### Recruitment

Individuals with PCS are recruited from the former ErgoLoCo study cohort for pragmatic reasons and because they have already experience and knowledge of occupational therapy and self-management approaches to FTG. All potential participants (*n* = 90) are invited to participate via email if they agreed to be re-contacted. If more than five individuals express interest, participants are randomly selected by lot. Occupational therapists are similarly recruited through the ErgoLoCo therapist network; if more than two express interest, random selection by lot determines participation. The development team is completed by 2–3 academic researchers with various professional backgrounds from the Department of General Practice at UMG, the Clinic of Rheumatology and Immunology at MHH, and the Regional Cooperative Rheumatism Centre of Lower Saxony.

##### Informed consent

Potential participants will first receive study information via email. In a follow-up phone call, eligibility criteria will be assessed, and all study procedures and evolving questions discussed. Following, participants provide written informed consent and return signed documents by mail. Co-researchers receive compensation (100€ per workshop session) for their participation [21].

#### Pilot study

For the pilot study, *n* = 20–30 participants not involved in the app development, with FTG and/or TC in the context of an underlying IRD, are to be enrolled. This sample size has been chosen pragmatically. Based on our experience from previous studies [15, 32] and recommendations from existing literature [33–35], we consider 20 participants to be the minimum and 30 participants to be the maximum that can be achieved with the project resources.

##### Eligibility criteria

The inclusion criteria for the pilot study are (1) a minimum age of 18 years; (2) self-reported symptoms of FTG and/or TC; (3) internet access; (4) an internet-enabled device; (5) German language proficiency; and (6) written informed consent to participate in the study. Potential participants who, due to the severity of their condition, are (1) unable to participate in sessions with an occupational therapist, or (2) a 1-h interview, or cannot independently test the app and provide written feedback, are not selected to participate for practical and ethical reasons.

##### Recruitment

Recruitment of participants for the pilot study will be conducted in cooperating rheumatology specialist practices in the German federal state of Lower Saxony. Leaflets and posters with information on study’s aims and procedures will be distributed and displayed in practice waiting rooms. Potential participants reach out to the study team by email or phone and are set on a waiting list until the pilot study begins. If more than 30 potential participants express interest and meet the inclusion criteria, a random selection process (by lot) will be conducted. If less than 20 participants are interested, recruitment efforts will be intensified, e.g., by including more practices or extending the recruitment period.

##### Informed consent

Prior to study start, potential participants on the waiting list will receive information on study’s aims, procedures, and data protection measures by email. In a follow-up phone call, eligibility criteria will be assessed and evolving questions discussed. After this, participants will provide consent by signing the consent form and mailing it to the study team. While app testing itself is not subject to compensation, participant will receive 80 Euros for participating in the follow-up interview following the pilot test phase.

### Data collection

Qualitative data will be collected throughout planning, development, and pilot testing phases to enable (1) systematic monitoring of the participatory process, (2) evaluation of app content and usability, and (3) critical reflection on co-researcher integration. All workshops and individual sessions will be conducted via Zoom, digitally recorded, and subsequently transcribed semantically by academic researchers [36]. Researchers will alternate moderation, co-moderation, and documentation responsibilities. Both individual and workshop sessions will follow moderation guidelines collaboratively developed within the development team. For this purpose, the academic researchers will first prepare a draft for the structure of the sessions, present it in the first workshop, and then adapt it to the needs and wishes of the co-researchers based on the principle of consensus [23].

An independent academic researcher (ISc) conducts passive participant observation throughout development workshops, systematically documenting team interactions, role negotiations, and engagement with technical platforms using structured observation protocols developed with external methodological experts. Additionally, written feedback notes from co-researchers and pilot participants complement recorded data.

Following development, ISc conducts semi-structured individual interviews to reflect the participatory development process. Interview guidelines are developed using the SPSS (Collect, Check, Sort, Subsume) method by Helfferich [37]. The aim is to create a guideline that provides a coherent structure for the interviews while allowing flexibility for unexpected topics to emerge.

During the testing of the DiEgO app by patients with FTG from the rheumatology practices, all participants (20–30) will be asked to provide written feedback on the intervention contents. For evaluation purposes, academic researchers will also conduct approximately 1-h semi-structured individual interviews with 12–15 patients after the testing phase. The interview guideline will be developed cooperatively by members of the development team, based on methodological literature and a guideline from ErgoLoCo. The sample size for qualitative evaluation is oriented to existing literature on good qualitative research practice, which recommends conducting at least 12 interviews [35]. Additionally, thematic saturation will be sought.

### Data analyses

In analyzing the observation protocols created for evaluating the participatory development process, the grounded theory approach by Glaser and Strauss [24] will be employed. This method is suitable for examining complex and dynamic processes with high interaction density because it offers both a clear methodological structure and flexibility. The material will be systematically analyzed in three steps.

#### Three steps of grounded theory according to Glaser and Strauss

- Step 1: Open coding: assign relevant phenomena to codes, condense codes into initial categories, and create notes or memos related to the research questions.
- Step 2: Axial coding: link the developed categories, focusing on aspects such as conditions, strategies, or consequences.
- Step 3: Selective coding: develop a core category representing the central phenomenon based on the codes.

Through this process, grounded theory enables the discovery of patterns in observations that can be subsequently compared [24]. After the analysis, interpretation of results will be conducted using Actor-Network Theory (ANT) by Latour [38]. ANT will specifically assist in theoretically contextualizing observations related to interactions between human and non-human actors (e.g., researchers and CIAS-EU).

The remaining data analysis will be based on Kuckartz’s structured qualitative content analysis [23]. This method was chosen because it is well-suited for qualitative research projects like this one, where extensive datasets require systematic content analysis with limited time and personnel resources. The structured content analysis by Kuckartz generally comprises seven phases. For data collected during the planning and development phase through introductory sessions, research workshops, and individual meetings, only the first analysis phase is planned, due to pragmatic and time constraints. This also applies to the written feedback from co-researchers and testers of the DiEgO app. Regarding the foundational data for the qualitative evaluation of the study gathered from individual interviews with development team members and testers of the DiEgO app, all seven analysis phases will be conducted.

#### Seven phases of content-structuring qualitative content analysis according to Kuckartz

- Phase 1: Initiating text work: highlight important text passages and write memos and case summaries.
- Phase 2: Develop thematic main categories: combine deductive and inductive categorization.
- Phase 3: First coding process: code the entire material using main categories.
- Phase 4: Compile all text passages coded with the same category.
- Phase 5: Inductive determination of subcategories within the material.
- Phase 6: Second coding process: code the complete material using the differentiated categories.
- Phase 7: Simple and complex analyses: evaluate categorically along main and subcategories (create thematic summaries, quantitative presentation, and qualitative interpretation of statements).

Data will be coded and analyzed by academic researchers using MAXQDA software, version 24.8.0 (VERBI Software GmbH, Berlin). To ensure validity and intercoder reliability, a consensual coding approach is employed, with researchers discussing results repeatedly [23]. After completing the seven phases of Kuckartz’s structured content analysis, the results will be processed, validated through circulation among co-researchers and DiEgO app testers, contextualized using empirical and theoretical literature, and documented in a results report.

## Discussion

DiEgO is a participatory research project that collaboratively develops an app-based intervention with occupational and exercise therapy content for individuals with FTG and TC. Qualitative methods are employed throughout the project to monitor the participatory development process, assess the intervention’s usability and acceptability through pilot testing, and reflexively evaluate how co-researchers are integrated into the research process. People with PCS and occupational therapists acting as co-researchers participate in nearly all phases of the research and development process. This ensures that the intervention being developed, along with its integrated content, is tailored to the needs of individuals affected by FTG and/or TC and adapted to the formal requirements of occupational therapy. By grounding development in lived experience and professional expertise, the participatory approach aims to enhance the intervention’s acceptability, feasibility, and suitability, thereby supporting well-being, quality of life, and social participation among individuals with FTG and TC.

A few studies have examined digital rehabilitation or self-management approaches developed for people with PCS and/or individuals with FTG [39–41]. However, these studies either only contain participatory elements such as advisory boards or multidisciplinary steering groups [39, 40], participation or patient and public involvement (PPI) only occurs in certain phases of the research process such as planning, data collection, or evaluation [39–41], or only interviews with patients or caregivers are conducted to better understand their perspectives [41] or to make adjustments after the initial development phase [40]. Unlike in this study, either no participatory approaches are used, or participation/PPI takes place only at the level of consultation [27].

The use of the open-source platform CIAS-EU enables collaborative development without programming expertise, allowing co-researchers to directly view and refine intervention content in real-time. This facilitates iterative adjustments based on immediate feedback from affected individuals and healthcare providers. The modular intervention design supports flexible application within professional occupational therapy, e.g., to supplement occupational therapy, for bridging until appointments are available, or for self-management.

Besides the benefits already mentioned, participatory research approaches also include some risks that sometimes are difficult to anticipate. For example, participatory research processes are only possible if there is trust between the participants. Establishing this trust requires careful planning of the collaboration in advance, moderation, and the continuous cultivation of a trusting working relationship, which is extremely time-consuming and costly and represents one of the greatest challenges of participatory research [27]. In many cases, this quality of cooperation is not achieved and instead often results in “functional participation,” which limits people to a supporting role and thus keeps them out of decision-making processes about the course of research [22]. In addition to the risk that a productive working relationship based on trust will not develop, there is a risk that the researchers will not agree on decision-making processes. Unlike in non-participatory research projects, there are no pre-defined hierarchies among the researchers in a participatory team, where, for example, the project leader makes important decisions. In participatory research projects, the project leader focuses on moderating joint decision-making processes within the research team [27]. However, the open dialogue on an equal footing that is necessary for this does not automatically lead to consensus and can result in different points of view on a health issue and thus in divergent actions. The wide variety of perceptions sought in participatory research projects can irritate those involved, as believed truths are called into question. Enduring and encouraging this irritation and the possible dialectical tensions that result from it to allow the “creative chaos” conducive to synthesis to emerge is another major challenge of participatory research approaches [27].

This study focuses on participatory intervention development and pilot testing, and thus does not seek to produce evidence on effectiveness. However, the iterative review by patients and healthcare providers provides insights into acceptability, feasibility, and suitability of the intervention, which will inform the design of future effectiveness trials. While development focuses on PCS, the intervention addresses FTG and TC broadly and may benefit individuals with other conditions such as ME/CFS, cancer, multiple sclerosis, or rheumatic diseases. Pilot testing with rheumatic patients will explore transferability.

Future publications from this study will reference this protocol. Consistent with participatory research principles, described methods and processes may be subject to adaption throughout the project to accommodate co-researcher needs, particularly those being personally affected by FTG and/or TC. Any fundamental changes will be incorporated into study protocol updates.

## Data Availability

No datasets were generated or analysed during the current study.

## Abbreviations

ANT: Actor-Network Theory
CIAS-EU: Computerized Intervention Authoring System (European Union version)
DiEgO: Digital Occupational Therapy for fatigue and trouble concentrating
ErgoLoCo: Occupational Therapy for Long COVID
FTG: Fatigue
GWDG: Society for Scientific Data Processing mbH Göttingen
IRD: Inflammatory rheumatic diseases
ME/CFS: Myalgic encephalomyelitis/chronic fatigue syndrome
MHH: Hannover Medical School
MS: Multiple sclerosis
MSU: Michigan State University
PCS: Post-COVID syndrome
PPI: Patient and public involvement
SPSS: Collect, Check, Sort, Subsume
UMG: University Medical Center Göttingen
